# Cancer of the Rectum and Pregnancy

**Published:** 1890-09

**Authors:** 


### CANCER OF THE RECTUM AND PREGNANCY.

---

Dr. Lohlein, of Giessen, having under his care a case of this kind, induced premature labor eight weeks before term. A fortnight later, the rectum was successfully excised.

In the *Zeitschrift fur Geburtschulfe*, Dr. Lohlein discussed the merits of the line of treatment which was pursued in this case. He maintained that waiting was wrong in any case of cancer. Operations on a pelvic organ during pregnancy entailed unusual danger from hemorrhage difficult to check under the circumstances. Abortion—a very probable result—would disturb the operation wound. Therefore, he held that when rectal cancer was diagnosed in a pregnant woman, labor should be induced, and an operation performed on the rectum as soon as possible. A serious accident occurred in connection with the case above mentioned. It caused a puerperal epidemic, whereby two new-born children died of erysipelas and a lying-in patient had severe fever, which, fortunately, did not prove fatal. This accident proved that, in lying-in hospitals, there may be other sources of infection besides the discharges, excreta, and exhalations of parturient women. New growths, beginning to break down, may escape the vigilance of trained midwives.—*British Medical Journal.*

